# Pharmacometabolomics identifies dodecanamide and leukotriene B4 dimethylamide as a predictor of chemosensitivity for patients with acute myeloid leukemia treated with cytarabine and anthracycline

**DOI:** 10.18632/oncotarget.20733

**Published:** 2017-09-08

**Authors:** Guangguo Tan, Bingbing Zhao, Yanqing Li, Xi Liu, Zhilan Zou, Jun Wan, Ye Yao, Hong Xiong, Yanyu Wang

**Affiliations:** ^1^ School of Pharmacy, Fourth Military Medical University, Xi’an, 710032, China; ^2^ Department of Hematology, The Central Hospital of Xuhui District, Shanghai, 20031, China

**Keywords:** pharmacometabolomics, metabolomics, acute myeloid leukemia, chemosensitivity, liquid chromatography-mass spectrometry

## Abstract

Clinical responses to standard cytarabine plus anthracycline regimen in acute myeloid leukemia (AML) are heterogeneous and there is an unmet need for biological predictors of response to this regimen. Here, we applied a pharmacometabolomics approach to identify potential biomarkers associated with response to this regimen in AML patients. Based on clinical response the enrolled 82 patients were subdivided into two groups: complete remission(CR) responders (n=42) and non-responders (n=40). Metabolic profiles of pre-treatment serum from patients were analyzed by ultra-high performance liquid chromatography coupled with mass spectrometry and the metabolic differences between the two groups were investigated by multivariate statistical analysis. A metabolite panel containing dodecanamide and leukotriene B4 dimethylamide (LTB4-DMA) had the power capacity to differentiate the two groups of patients, yielding an area under the receiver operating characteristic of 0.945 (85.2% sensitivity and 88.9% specificity) in the training set and 0.944(84.6% sensitivity and 80.0% specificity) in the test set. The patients with high levels of LTB4-DMA and low amounts of dodecanamide had good sensitivity to chemotherapeutic agents. The possible reasons were that dodecanamide was produced by leukemic cells as a lipolytic factor to fuel their growth with a potential role in drug resistance and LTB4-DMA was a potent leukotriene B4 antagonist that could be applicable in the treatment of AML. These preliminary results demonstrates the feasibility of relating chemotherapy responses with pre-treatment metabolic profiles of AML patients, and pharmacometabolomics may be a useful tool to select patients that are more likely to benefit from cytarabine plus anthracycline chemotherapy.

## INTRODUCTION

Acute myeloid leukemia (AML) is a clinically and biologically heterogeneous hematologic malignancy that is standardly treated with combinations of cytarabine and anthracycline [1]. The scheme regimen can significantly benefit most of the patients; however, the heterogeneous response to such therapy demonstrated that 30-40% of the patients suffer from adverse effects without any positive results [2], thereby losing the chance of trying alternative chemotherapy if their physical condition has deteriorated too far. Hence, an ability to predict the response to chemotherapeutic agents has an important implication in developing personalized treatment strategies, improving survival rates and reducing unnecessary exposure of patients to toxic drugs.

Currently, research focused on finding useful molecular or clinical predictors of chemosensitivity in AML is relatively sparse. Genome-wide complementary DNA microarray analysis has been explored to find gene signatures associated with chemosensitivity, in which a “Drug Response Scoring” system with sensitivity of 85% was developed based on twenty-eight difference genes between good and poor responders to chemotherapy [3]. However, this technique was somewhat limited by the high costs. Proteomics was proposed to predict clinical responses to chemotherapy. It has been shown that the peak at *m/z* 6611 from the weak cation exchange pH 9 fraction, when combined with age, provided strong positive prediction of responders with 83% accuracy [4]. In addition, several pharmacogenetic studies have been explored to find the impact of polymorphisms in genes encoding transporters, metabolizers or molecular targets of chemotherapy agents such as cytarabine and anthracycline [5]. Other molecular markers such as FCHSD2 [6], nucleosomal DNA fragments [7] and bone marrow MicroRNA-335 [8] have also been identified as potential predictors of chemotherapy response. However, suboptimal performance is a major issue that limits their wide applicability.

Metabolic profiling (metabonomics/metabolomics) based on nuclear magnetic resonance (NMR) and mass spectrometry (MS), an alternative strategy for biomarker discovery, enables identification of small-molecule metabolites in biofluids and tissues that are sensitive to altered pathology [9], because a minor alteration at the level of gene or protein expression usually results in a significant change in small molecule metabolite level [10]. In the past few years, metabolomics approaches have been widely used in cancer detection, progression, and drug discovery [11, 12]. Recently, the pretreatment biofluid metabolomic profiles (Pharmacometabolomics) have also been successfully applied to predict the metabolic fate and toxicity of drugs and the response to neoadjuvant chemotherapy [13–19]. Compared with other biomarker discovery approaches for AML, metabolomics provides a strong link between genotype and phenotype [20], and may provide some insight into the pathological state of the disease, which is believed to be an alternative strategy for individualized therapy of cancer.

Until now, several metabolomics studies are contributing toward an improved understanding of AML, and these advances have been reviewed [21]. AML prognostic factors, such as 2-hydroxyglutarate and glucose metabolism signature included a group of six metabolite biomarkers [22, 23], could be predicted by gas chromatography- mass spectrometry (GC-MS) based metabolomics on serum samples. In another GC-MS study, it was demonstrated that fatty acid metabolism was deregulated in patients with AML and might represent an underlying metabolic pathway associated with disease progression [24]. A recent cellular metabolomic study with liquid chromatography-mass spectrometry (LC-MS) showed that resistant leukemia cells exhibit reduced glutamine dependence, enhanced glucose dependence, and altered fatty acid metabolism [25]. In this study, we use pharmacometabonomic approach based on ultrahigh performance liquid chromatography (UHPLC) coupled with Q-TOF mass spectrometry to predict the response to chemotherapy for *de novo* AML patients treated with cytarabine and anthracycline. The response of patients with AML to chemotherapy could be differentiated based on serum metabolite profiles obtained prior to initiation of cytotoxic therapy. We found that patients with lower amounts of dodecanamide and higher levels of leukotriene B4 dimethylamide (LTB4-DMA) responded more successfully to the treatment. A statistical model built on the two metabolites predicts response to chemotherapy with high sensitivity and specificity. We expected that the pharmacometabonomic approach could be conveniently applied to other anticancer agents and contribute to improving chemotherapy of cancer.

## RESULTS

### Quality control of the methodologies

**Figure 1:**
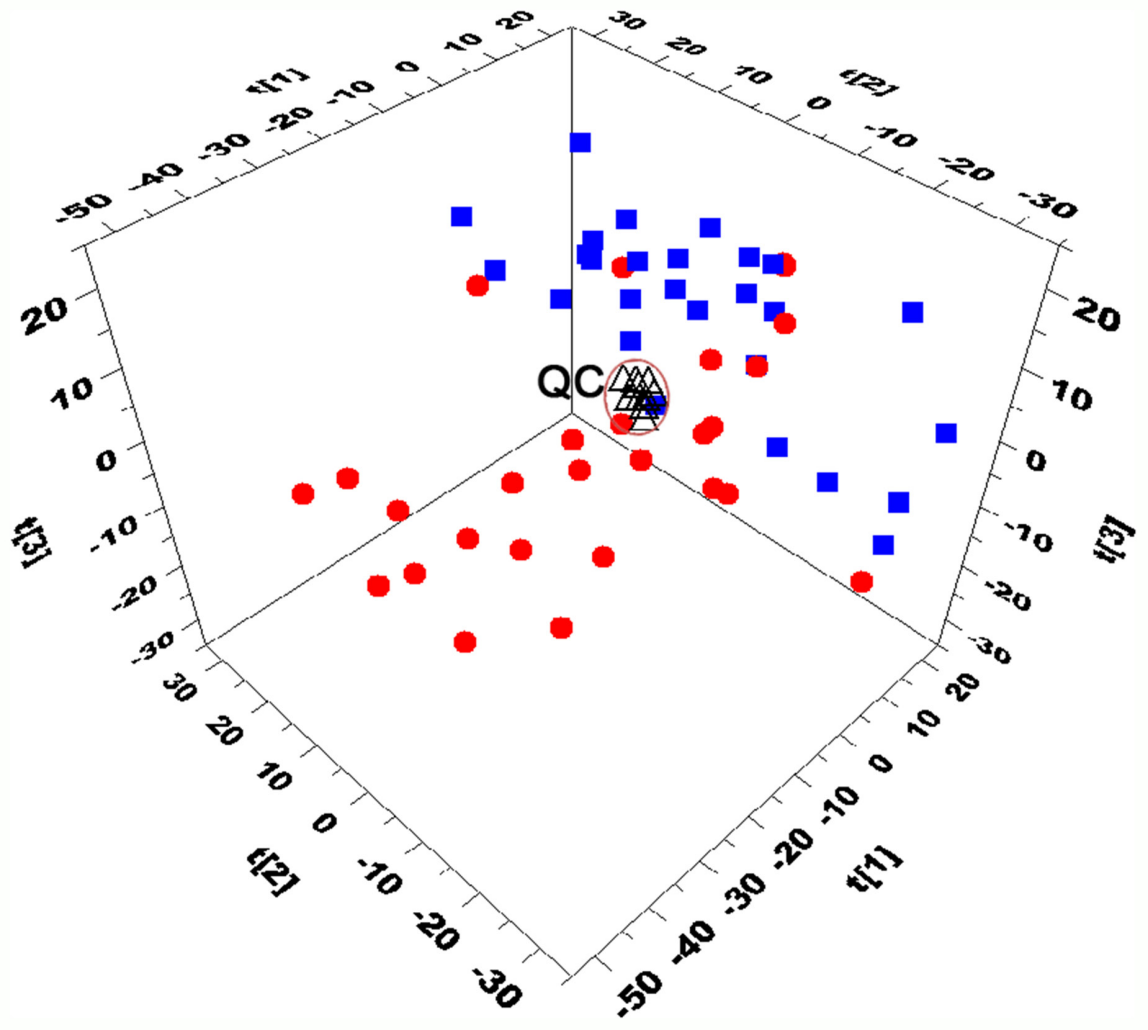
Three-dimensional PCA score plots based on the data from UHPLC-Q-TOFMS separation (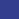
CR patients, 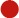
NR patinets, ΔQC) The QC cluster is highlighted within the black ellipses.

The stability of the analytical method is very important to obtain valid data that can display the biochemical snapshot. Chromatograms obtained from the real samples and QCs were aligned together and filtered to obtain features with relative standard deviations (RSDs) less than 30% in QCs and present in more than 80% of QCs. Finally, a dataset with 1439 features was produced, covering 87.3% features in UHPLC–QTOF-MS analysis. The result indicated that the present method had good repeatability. In addition, PCA was used to provide an overview of the training set samples and QCs after unit variance scaling. As shown in Figure 1, the close clustering of QC samples are observed, reflecting the excellent stability of analytical system and the reproducibility of the sample preparation procedure.

### Differentially expressed metabolites between CR and NR serum samples

**Figure 2:**
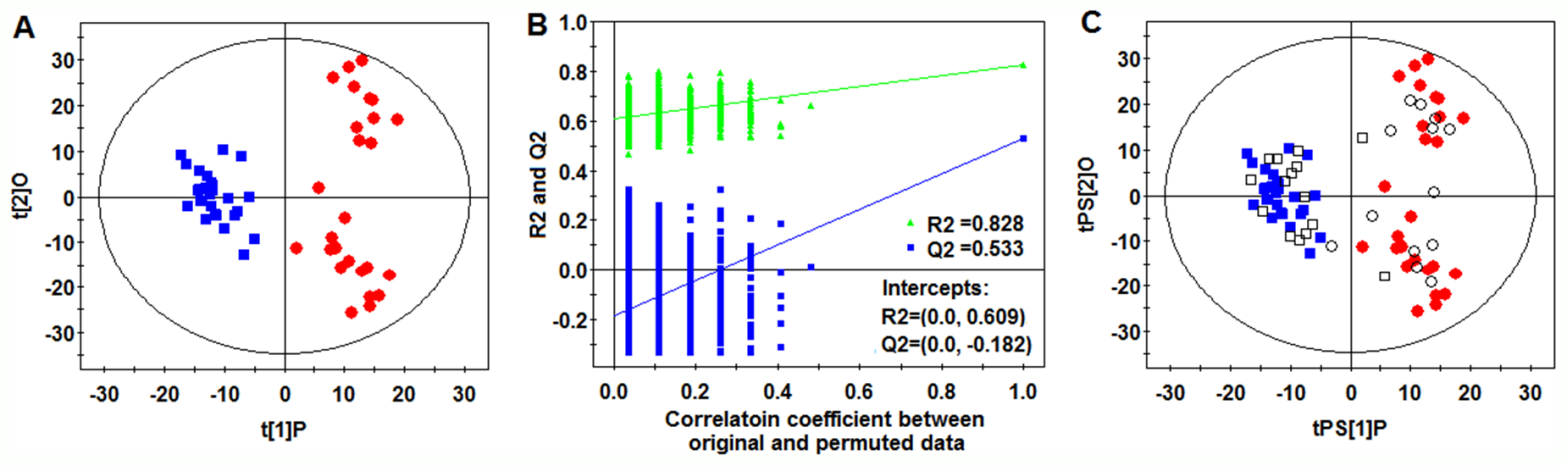
OPLS-DA analysis **(A)** OPLS-DA score plot. **(B)** Validation plot of the model obtained from 999 permutation tests (R^2^=0.828 and 0.609: the fraction of the Sum of Squares (SS) in the original and permuted data explained by the models, respectively. Q2=0.533 and -0.182: the cumulative cross validated R^2^ in the original and permuted data, respectively). **(C)** T-predicted scatter plots of the OPLS-DA model(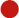
CR patients, 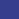
NR patinets, □ CR patients prediction set, ○NR patients prediction set).

PCA scores plot revealed a trend of separation between CR and NR samples of the training set (Figure 1). To further identify metabolites that can discriminate between CR and NR groups, the supervised OPLS-DA model was established in that it was more focused on the actual class discriminating variation compared to the unsupervised PCA model. An OPLS-DA model was obtained with one predictive component and three orthogonal components (R^2^Xcum=0.451; R^2^Ycum=0.919; and Q^2^cum=0.672). A clear separation between CR group and NR group was observed in the OPLS-DA scores plot by the first two components (Figure 2A). To validate the model, a permutation test with 999 iterations were performed. In the model, R^2^ is defined as the fraction of the Sum of Squares (SS) in the data explained by the models and indicates goodness of fit. Q^2^ is defined as the fraction of the SS in the data predicted by the model and indicates predictability, calculated by a cross validation procedure. By comparing the R^2^ and Q^2^ of the original model with the ones of randomly permuted models, we could evaluate the fitness and prediction ability of the models [26]. As shown in Figure 2B, the validation plot strongly indicates that the original model is valid. The criteria for validity are: all the permuted R^2^ and Q^2^ values to the left are lower than the original point to the right and the blue regression line of the Q^2^ (cum) points has a negative intercept [26]. To further evaluate the predictive ability of the established models, an independent validation set consisting of 28 samples (collected from 15 CR AML patients and 13 NR AML patients) was performed. None of those samples had been previously included in the supervised analysis, which therefore allowed for the estimation of true predictive accuracy. As shown in Figure 2C, the *T* predicted score plot of OPLS-DA demonstrated that 3 out of 28 samples are wrongly assigned in the direction of the first principal component, implying that 89.3% of the samples are predicted correctly. This external validation study confirms the feasibility of UHPLC-MS-based serum metabolic profiles as a potential predictor for chemosensitivity.

Variables (metabolites) that significantly contributed to the clustering and discrimination were identified according to a threshold of variable importance in the projection (VIP) values (VIP>1), which could be generated from OPLS-DA model. In order to select potential biomarkers worthy of preferential study in the next step, these differential metabolites were validated using Student’s *t* test and fold change. The critical *p*-value was set to 0.05 for significantly differential variables and the fold change was set to 1.4 in this study. Following the criterion above, a number of 10 metabolites responsible for discriminating between CR and NR groups were identified (Table 1). The extracted ion chromatograms (EICs) from two representative samples (one from CR serum and one from NR serum) were provided in Supplementary Figure 1, in which the 10 metabolite markers were marked. The details of the fragments in each MS/MS spectrum for each identified metabolite are presented in Supplementary Figure 2.

**Table 1:** Differential metabolites for discrimination between CR AML patients and NR AML patients

No.	m/z	rt(min)	Formula	Metabolite^*a*^	FC^*d*^	VIP^*e*^	p value ^*f*^	*q* value	AUC^*g*^	sensitivity(%)	specificity(%)
1	137.0455	1.04	C_5_H_4_N_4_O	Hypoxanthine^*b*^	0.67	1.19	6.79×10^-3^	1.01×10^-2^	0.72(0.58-0.84)	0.70	0.63
2	166.0862	2.14	C_9_H_11_NO_2_	Phenylalanine^*b*^	0.69	1.02	3.34×10^-2^	3.21×10^-2^	0.64(0.50-0.75)	0.67	0.59
3	172.1694	9.11	C_10_H_21_NO	Decanamide^*b*^	1.55	1.47	6.28×10^-4^	1.90×10^-3^	0.78(.065-0.88)	0.59	0.78
4	300.2894	9.88	C_18_H_37_NO_2_	Sphingosine^*b*^	1.49	1.38	1.50×10^-3^	3.47×10^-3^	0.75(0.61-0.86)	0.63	0.74
5	200.2008	10.58	C_12_H_25_NO	Dodecanamide^*b*^	1.70	2.22	1.89×10^-8^	<1.0×10^-8^	0.90(0.79-0.97)	0.70	0.81
6	364.2845	12.28	C_22_H_37_NO_3_	Leukotriene B4 dimethylamide^*b*^	0.65	2.37	6.84×10^-10^	<1.0×10^-8^	0.92(0.82-0.98)	0.78	0.88
7	287.2218	12.89	C_16_H_30_O_4_	Hexadecanedioic acid^*c*^	1.41	1.62	1.33×10^-4^	<1.0×10^-8^	0.76(0.62-0.86)	0.67	0.78
8	674.4632	13.52	C_36_H_68_NO_8_P	PC(14:1(9Z)/14:1(9Z))^*c*^	0.68	1.36	1.75×10^-3^	3.47×10^-3^	0.76(0.62-0.86)	0.67	0.63
9	282.2791	13.66	C_18_H_35_NO	Oleamide^*b*^	1.49	1.34	2.18×10^-3^	3.47×10^-3^	0.76(0.62-0.86)	0.56	0.74
10	554.5509	13.79	C_35_H_71_NO_3_	Cer(d18:0/17:0)^*c*^	0.71	1.39	1.42×10^-3^	1.90×10^-3^	0.76(0.63-0.87)	0.67	0.81

^a^The metabolites marked with“*c*” were putatively annotated, the metabolites marked with “*b*” were structurally identified by reference standards. ^*d*^ Fold change was calculated from the normalized peak area between NR group vs GR group.^*e*^ Variable importance in the projection (VIP) was obtained from the OPLS-DA model.^*f*^ The p value was calculated from Student’s t test.^*g*^ Area under the receiver operating characteristic (ROC) curve, with the 95% confidence interval (CI) range in parentheses.

### A metabolite panel for chemosensitivity

The performance of these metabolites for chemosensitivity was evaluated individually using receiver operating characteristic (ROC) curves and sensitivity as well as specificity. Table 1 list the area under the ROC (AUC) values, sensitivity and specificity. Dodecanamide, LTB4-DMA and cer(d18:0/17:0) showed the specificity of greater than 0.80 in classifying NR and CR patients. However, none of the 10 metabolites distinguished CR from NR patients with both sensitivity and specificity of greater than 0.80, which making it necessary to apply multiple serum metabolites in the discrimination of CR AML patients out of all AML patients. AML generally contained systematic dysregulation of multiple metabolic pathways, and the chemotherapy of AML was also involved in multiple pathways. Therefore, a panel of metabolites had more prediction power for the chemosensitivity of AML than one metabolite.

**Figure 3:**
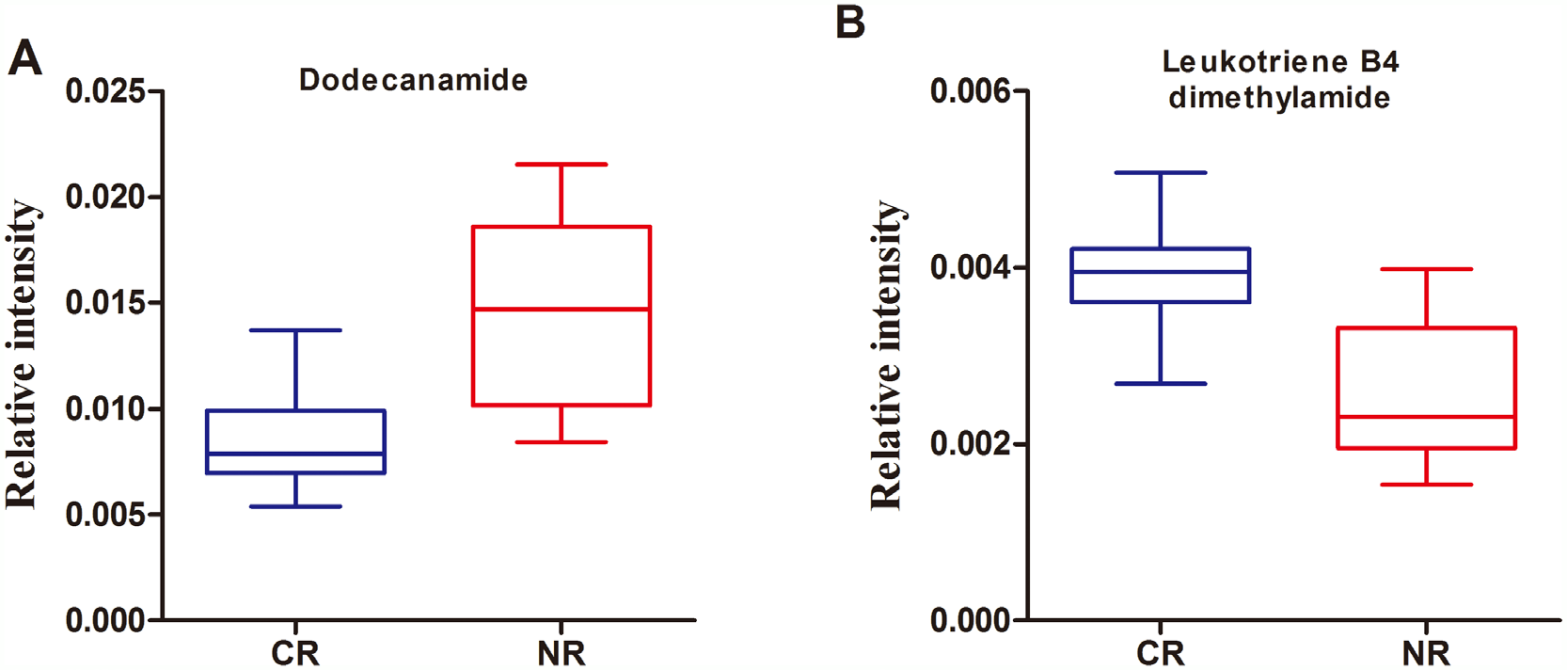
Box-and-whisker plots of the normalized peak areas of dodecanamide **(A)** and leukotriene B4 dimethylamide **(B)**.

To identify a metabolite panel associated with chemosensitivity of AML, a binary logistic regression model with a stepwise optimization algorithm was performed, which is used to model relationships between a dichotomous dependent variable (CR/NR) and multiple independent variables (10 differential metabolites). This analysis showed that two metabolites including dodecanamide and LTB4-DMA could be attributed to the most significant deviations between NR and CR groups, indicating that these two metabolites produced the better predictive power for future chemosensitivity applications. The box-and-whisker plots for the relative concentrations of these two metabolites are presented in Figure 3. The prediction model is as follows: P =1/[1+exp(−(1.421+558.482×(dodecanamide)−2141.843×(LTB4-DMA)))].

**Figure 4:**
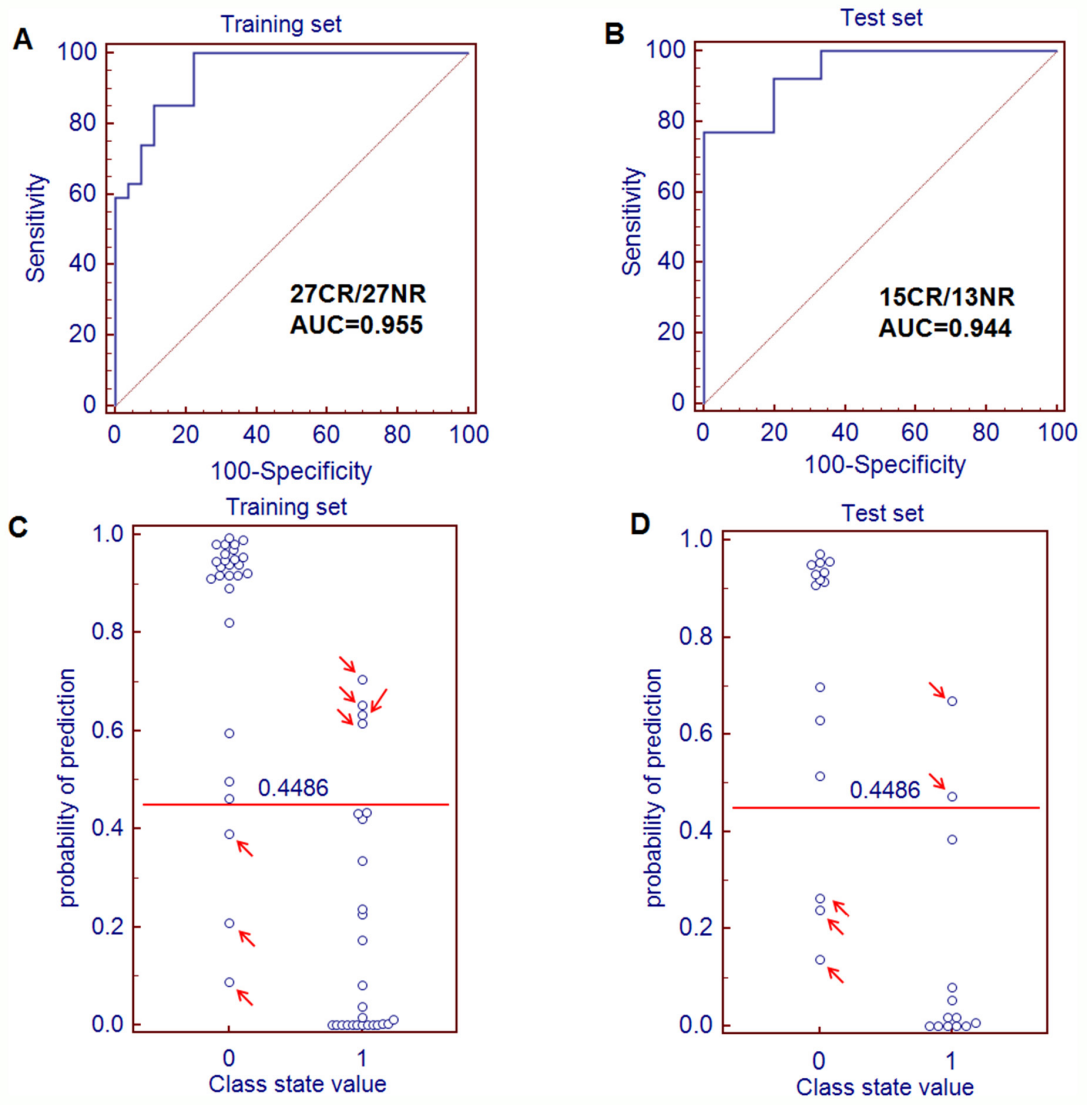
Quantification of the diagnostic performance of the metabolite panel containing dodecanamide and leukotriene B4 dimethylamide and the prediction plots according to the optimal cutoff value obtained from ROC curves **(A)** The ROC curves of the training set (A) and test set **(B)** were obtained from the prediction model. The optimal cutoff value was obtained (0.4486) and applied to evaluate the prediction capacity (87.0% for training set **(C)** and 82.1% for test set **(D)** of the current model, where 0 and 1 on the x axis represent CR AML patients and NR AML patients, respectively, and blue circle represent samples.

The ROC curve was further conducted to evaluate the performance of this prediction model. It demonstrated that a metabolite panel containing the two metabolites yielded an AUC of 0.945 ( 85.2% sensitivity and 88.9 specificity, Figure 4A). Based on this sensitivity and specificity of the ROC curves in the training set, an optimal cutoff value of 0.4486 was produced. If the probability of prediction calculated from the prediction model was more than the cutoff value of 0.4486, the subject could be diagnosed as a chemotherapy-sensitive patient. Otherwise, the subject could be diagnosed as a nonsensitive patient. On the basis of this cutoff value, it was observed that 47 out of 54 patients (87.0%) could be accurately predicted the chemosensitivity (Figure 4C), which demonstrated that the response to chemotherapy of patients with AML could be well-stratified by using the combination of dodecanamide and LTB4-DMA in the training set.

### Validation of the metabolite panel for chemosensitivity

Based on the training set, the metabolite panel was identified and preliminarily validated as a good predictor for chemosensitivity. In order to validate this metabolite panel before proceeding to a larger-scale clinical trial, the metabolite panel was used to classify blinded diverse samples from an independent test cohort of 15 CR patients and 13 NR patients. ROC analysis yielded an AUC of 0.944 (84.6% sensitivity and 80.0% specificity; Figure 4B) in discriminating NR patients from CR patients to chemotherapy. On the basis of the cutoff value of 0.4486 from the training set, it was also found that 23 out of 28 samples (82.1%) in test set could be accurately predicted (Figure 4D).

## DISCUSSION

In this study, we present a new metabolomics approach for predicting chemotherapy response in patients with AML. Although the clinical parameters of the patients with AML are remarkably heterogeneous, the serum metabolic profiles from patients still fall into distinct clusters which were related with their different responses to chemotherapy with cytarabine and anthracycline. To the best of our knowledge, this is the first study to report the predictive capacity of metabolomics to allow identification of response to chemotherapy using pretreatment serum samples from patients with AML. Ten metabolites related to chemosensitivity were identified in this study. Unfortunately, none of the metabolites exhibited both high sensitivity and high specificity. AML generally has systematic dysregulation of several metabolic pathways and therefore multiple baseline metabolites will show a more robust capacity to predict chemosensitivity. By constructing a binary logistic regression model, a metabolite panel containing dodecanamide and LTB4-DMA was shown to be highly correlated with chemosensitivity, yielding an AUC of 0.945 (85.2% sensitivity and 88.9% specificity) in the training set and 0.944(84.6% sensitivity and 80.0% specificity) in the test set.

As shown in Figure 3A, the dodecanamide levels in NR group was higher than those of CR group. Among the 10 potential biomarkers identified, dodecanamide, decanamide, and oleamide were fatty acid amides and the significantly higher levels of them were observed in NR patients relative to CR patients. Dysregulated lipid metabolism is a feature of cancer and there is growing evidence for the importance of fatty acid amides in AML biology [27]. Peroxisome proliferator activated receptor (PPAR) could be activated by fatty acid amides and was thought to aid in leukemic cell survival against cytotoxic stressors such as chemotherapeutic drugs [28]. For example, oleoylethanolamide, an endogenous PPAR-α agonist, was significantly high level in the plasma of chronic lymphocytic leukemia patients such that its plasma concentration was directionally related to the number of circulating leukemic cells [29]. In that study, it was suggested that oleoylethanolamide was produced by leukemic cells as a lipolytic factor to fuel their growth with a potential role in drug resistance and cancer cachexia [29], which could give an clue to the physiological functions of dodecanamide, decanamide, and oleamide in this study. This was the possible reason that the AML patients with a low level of dodecanamide have better chemosensitivity than those with a high level of dodecanamide.

The performance of LTB4-DMA was particularly striking, and showed an AUC of 0.92 for the classification of CR from NR patients. LTB4-DMA is a derivative of Leukotriene B4 (LTB4). Leukotrienes (LTs) are biologically active metabolites of arachidonic acid *via* 5-lipoxygenase (5-LO) in the body by myeloid cells and B lymphocytes [30]. A recent study was proposed that LTB4 plays an important role in AML cell activation and proliferation, and it indicated that leukotriene biosynthesis inhibitors or antagonists similar to those employed in the treatment of asthma could be applicable in the treatment of AML [31]. Our data demonstrated that the level of LTB4-DMA in the NR AML patients was lower than that of CR AML patients, as shown in Figure 3B. Although the mechanism that regulated the endogenous formation of LTB4-DMA in AML patients was not yet known, a accumulating evidence that LTB4-DMA was a potent leukotriene B4 antagonist [32]. Thus, it was speculated that LTB4-DMA increased the chemosensitivity of AML in that the endogenous LTB4-DMA may have a therapeutic role in AML.

Taken together, this study, although on a relatively small cohort of AML cases, represents ‘proof of principle’ and demonstrates the feasibility of relating chemotherapy responses with the pretreatment serum profiles of AML patients during treatment with standard cytarabine and anthracycline-based induction chemotherapy. The combination of serum dodecanamide and LTB4-DMA given an effective predictor for chemosensitivity. ROC analysis shows that the biomarker pattern achieves a sensitivity and specificity more than 80%. This potential specific biomarker pattern may thus be an alternative method to the prediction of chemosensitivity for AML patients. Further study toward clinical applications is under consideration as possible extensions of our work. The implementation of the metabolite panel containing dodecanamide and LTB4-DMA should undergo a strict process of initial quantitation assay establishment, and multi-center cross-validation. Therefore, consolidation studies on a larger number of AML patients are needed and ongoing.

## MATERIALS AND METHODS

### Chemicals and reagents

HPLC-grade methanol and acetonitrile (ACN) were purchased from Merk (Darmstadt, Germany). Formic acid was obtained from Fluka (Buchs, Switzerland). LTB4-DMA purchased from Abcam (Cambridge, UK). Sphingosine, decanamide, and oleamide were purchased from Sigma-Aldrich (St. Louis, MO). Hypoxanthine, phenylalanine and *L-*2-chlorophenylalanine (internal standard) were obtained from Shanghai Jingchun Reagent Co. Ultrapure water was prepared with a Milli-Q water purification system (Millipore, Bedford, MA, USA).

### Patients and sample collection

The *de novo* AML patients without severe heart, liver, or renal dysfunction were recruited from the Central Hospital of Xuhui district of Shanghai between May 2013 and May 2014. All patients provided informed consent in accordance with the University and Institutional Review Boards requirements and the Declaration of Helsinki. The diagnosis was made according to the French-American-British (FAB) classification and samples examined in this study were classified from M0 to M5 [33]. FAB-M3 samples were not included in this study because most of those patients were treated by more effective chemotherapy with *all-trans* retinoic acid. The cytogenetic risk was classified into three categories: favorable, intermediate, and unfavorable [34].

All patients received the same induction therapy with cytarabine at 100 mg/m^2^/d by continuous infusion for 7 days (day 1 to 7) and idarubicin at 8 mg/m^2^/d from day 1 to 3. Patients who achieved complete remission received two consolidation courses based on high-dose cytarabine at 2g/m^2^ during one 1-hour infusion every 12 hours for 4 days. With regard to therapy response, it was differentiated between patients with complete remission (CR group) and those with failure (non-responder group) of therapy. According to European LeukemiaNet criteria [34], complete remission was defined as less than 5% blasts in a normocellular marrow and peripheral blood counts showing ≥1×10^9^/L neutrophils and ≥100 ×10^9^/L platelets, without evidence of extramedullary leukemia. Patients with insufficient decline of the blasts, death earlier than 7 days after the end of the first induction cycle or death because of the treatment-induced bone marrow hypoplasia after chemotherapy were categorized as therapeutic failure. The patients were divided into a training set and a test set. The training set, composed of 27 CR responders and 27 non-responders (NRs), was used to find serum metabolites associated with chemosensitivity; the remaining subjects including 15 CR responders and 13 non-responders were used to construct the test set to independently verify the prediction biomarkers. Demographic and clinical data are listed in Table 2. Blood samples from 82 AML patients were collected prior to initiation of cytotoxic therapy. The collected blood was allowed to clot for 45 min at room temperature and centrifuged for 10 min at 3000 rpm at room temperature; the upper serum phase was then isolated, aliquoted and frozen at -80°C until further use.

**Table 2:** Detailed patient characteristics before the start of treatment

Characteristics	Training set			Test set		
	CR	NR	p value	CR	NR	p value
Size	27	27		15	13	
Age (years), median(range)	45 (17-70)	48 (15-71)	0.75	47(20-67)	46 (21-69)	0.85
Gender (male/female)	17/10	18/9		8/7	9/4	
FAB subtype, n(%)						
M2	6(22.2%)	5(18.5%)		2(13.3%)	2(15.4%)	
M4	13(48.2%)	14(51.9%)		8(53.3%)	7(53.8%)	
M5	8(29.6%)	8(29.6%)		5(33.4%)	4(30.8%)	
Cytogenic risk group, n(%)						
Favorable	4(14.8%)	2(7.4%)		2(13.3%)	1(7.7%)	
Intermediate	16(59.3%)	17(63.0%)		9(60%)	8(61.5%)	
Unfavorable	7(25.9%)	8(29.6%)		4(26.7%)	4(30.8%)	
WBC (10^9^/L), median(range)	12.5(1.1-90.0)	17.4(1.4-105.4)	0.67	39.0(1.6-149)	29.8(3.5-101)	0.76
LDH(U/L), median(range)	293(1.4-1279)	371(38-4147)	0.25	344(131-1529)	406(45-714)	0.98
Hemoglobin(g/L), median(range)	69(36-118)	64(21-108)	0.38	71(24-109)	68(30-110)	0.87
Platelet (10^9^/L), median(range)	36(5-383)	41(4-345)	0.67	50(4.6-423)	68(3.4-540)	0.58
BM Blast (%),	63(13-89)	67(14-90)	0.75	70(21-85)	52(12-90)	0.52

p values were calculated by means of Mann-whitney test. FAB, French-American-British; WBC, white blood cells;LDH, lactate dehydrogenase;BM blast: the percentage of blasts in bone marrow.

### UHPLC-Q-TOFMS analysis

Frozen serum samples (100 μL) were thawed and vortexed for 5 s at room temperature, and protein was precipitated by adding 400 μL methanol containing 12.5 μg/ml *L*-2-chlorophenylalanine as the internal standard to monitor extraction efficiency. The solutions were centrifuged at 14,000×g for 15 min at 4°C, the resulting supernatants were transferred into an auto-sampler vial. The concept of quality control samples (QCs) reported by Gika *et al.* was adopted here to monitor and evaluate the stability of the analysis [35]. A pooled sample, which was a mixture of small random volumes from all 82 samples, was extracted using the same procedure as above. This sample was used as a quality control (QC) and was analyzed after every 8 serum samples. UHPLC-Q-TOFMS analysis was performed using an Agilent 1290 Infinity LC system (Agilent, Germany) coupled with an Agilent 6530 Accurate-Mass Quadrupole Time-of-Flight (Q-TOF) mass spectrometer (Agilent, USA). Chromatographic separation was carried out at 40 °C on an ACQUITY UPLC HSS T3 C_18_ column (2.1mm ×100mm, 1.7 μm, Waters, Milford, MA). The column oven was set at 40 °C. The mobile phase consisted of 0.1% formic acid in water (A) and ACN modified with 0.1% formic acid (B), using a gradient elution of 5%B at 0–2 min, 5%–95% B at 2–13 min, 95% B at 13–15 min. The total run time was 20 min including equilibration. The flow rate was 350 μL/min and the injection volume was 4 μL. The Q-TOF mass spectrometer was operated in electrospray ionization source (ESI) positive ion mode with a capillary voltage of 3.5 kV, drying gas flow of 11 L/min, and a gas temperature of 350 °C. The nebulizer pressure was set at 45 psig. The fragmentor voltage was set at 120 V and skimmer voltage was set at 60 V. Data were collected in centroid mode and the mass range was set at *m/z* 50–1000 using extended dynamic range. Potential biomarkers were analyzed by MS/MS. MS spectra were collected at 2 spectra/s, and MS/MS spectra were collected at 0.5 spectra/s, with a medium isolation window (∼4 *m/z*) and a fixed collision energy of 10 V.

### Data handing and statistical analysis

The acquired UHPLC-Q-TOFMS data were exported in mzData format and then processed by XCMS package (http://metlin.scripps.edu/download/) as described in a previous publication [36]. Sample information, peak retention time, peak m/z, and peak area (quant mass) were included in the final dataset. The resulting data were normalized to the internal standard before statistical analysis. After mean-centering and unit variance (UV)-scaled for equal peak weighting, the normalized data was analyzed by principal component analysis (PCA) and orthogonal projection to latent structures-discriminant analysis (OPLS-DA), a regression extension of PCA, using SIMCA-P software (version 11.0; Umetrics). The default 7-fold cross-validation was applied, to guard against overfitting. The variable importance in the projection (VIP) values (VIP > 1.0) are considered to be differentiating variables. The Student t test was used for further differentiating variables selection and validation (P < 0.05). To account for multiple comparisons, false discovery rate was estimated as the maximum *q* value [37] in the set of significant differences for the metabolomic data set. False discovery rates were computed using the R package q value (http://www.r-project.org/). The software MedCalc (version 11.4.2.0) was used to perform variable selection of potential biomarkers and receiver operating characteristic (ROC) analysis based on binary logistic regression model. In addition, the patient characteristics were compared using the Mann–Whitney U test for continuous variables.

### Metabolite identification

Metabolite identification was carried out according to the authors’ previous work with slight modification [38]. Briefly, ions of interest were scanned in both positive and negative modes to facilitate the judgment of quasi-molecular ions. Potential molecular formulae were calculated by MassHunter Workstation Software-Qualitative Analysis (Agilent Technologies, California, United States). Structure information was obtained by searching freely accessible databases of HMDB (www.hmdb.ca), METLIN (http://metlin.scripps.edu) and KEGG (http://www.kegg.jp) utilizing detected molecular weights (under the above mentioned conditions, the mass difference was less than 10 ppm). At the same time, fragment ions were subjected to analysis through MS/MS to narrow the scope of target compounds. Finally, these metabolites were structurally confirmed by comparing the retention times and MS/MS spectra to the commercial standards.

## SUPPLEMENTARY MATERIALS FIGURES


