# Supplementary material for: Pharmacometabolomics identifies dodecanamide and leukotriene B4 dimethylamide as a predictor of chemosensitivity for patients with acute myeloid leukemia treated with cytarabine and anthracycline
Source: Oncotarget. 2017 Sep 8;8(51):88697–707. doi: 10.18632/oncotarget.20733 (PMC5687638; doi:10.18632/oncotarget.20733)
Supplement: Supplementary file 1 [file oncotarget-08-88697-s001.pdf]

Pharmacometabolomics identifies dodecanamide and leukotriene B4 dimethylamide as a predictor of chemosensitivity for patients with acute myeloid leukemia treated with cytarabine and anthracycline

SUPPLEMENTARY MATERIALS

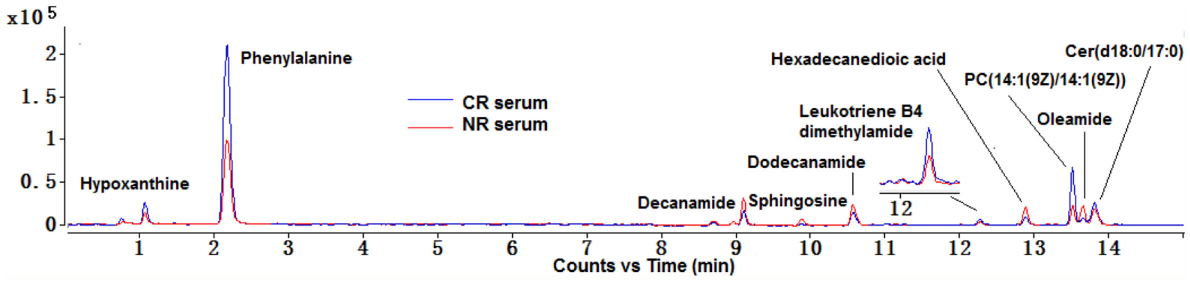

Supplementary Figure 1: Representative extracted ion chromatograms (EICs) obtained from one NR AML patients (red line) and one CR AML patients (blue line).

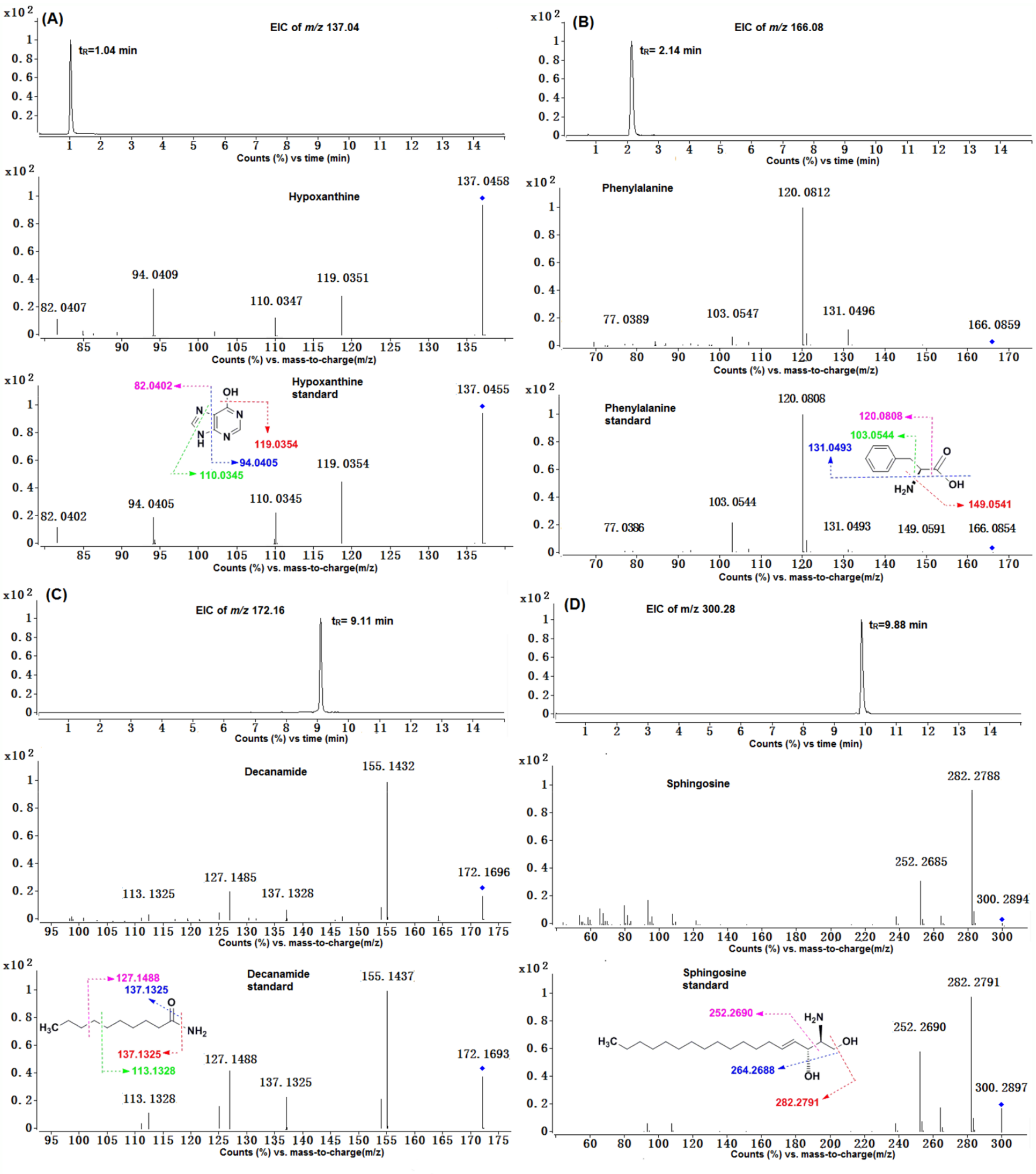
(A)
EIC of m/z 137.04
tR=1.04 min
Counts (%) vs time (min)
Hypoxanthine
137. 0458
94. 0409
119. 0351
110. 0347
82. 0407
Counts (%) vs. mass-to-charge(m/z)
Hypoxanthine standard
137. 0455
82.0402
119.0354
94.0405
110.0345
119. 0354
94. 0405
110. 0345
82. 0402
(B)
EIC of m/z 166.08
tR= 2.14 min
Phenylalanine
120. 0812
77. 0389
103. 0547
131. 0496
166. 0859
Phenylalanine standard
120. 0808
120.0808
103.0544
131.0493
149.0541
103. 0544
77. 0386
131. 0493
149. 0591
166. 0854
(C)
EIC of m/z 172.16
tR= 9.11 min
Decanamide
155. 1432
127. 1485
113. 1325
137. 1328
172. 1696
Decanamide standard
155. 1437
127.1488
137.1325
137.1325
113.1328
127. 1488
137. 1325
113. 1328
172. 1693
(D)
EIC of m/z 300.28
tR=9.88 min
Sphingosine
282. 2788
252. 2685
300. 2894
Sphingosine standard
282. 2791
252.2690
252. 2690
264.2688
282.2791
300. 2897

(*Continued*)

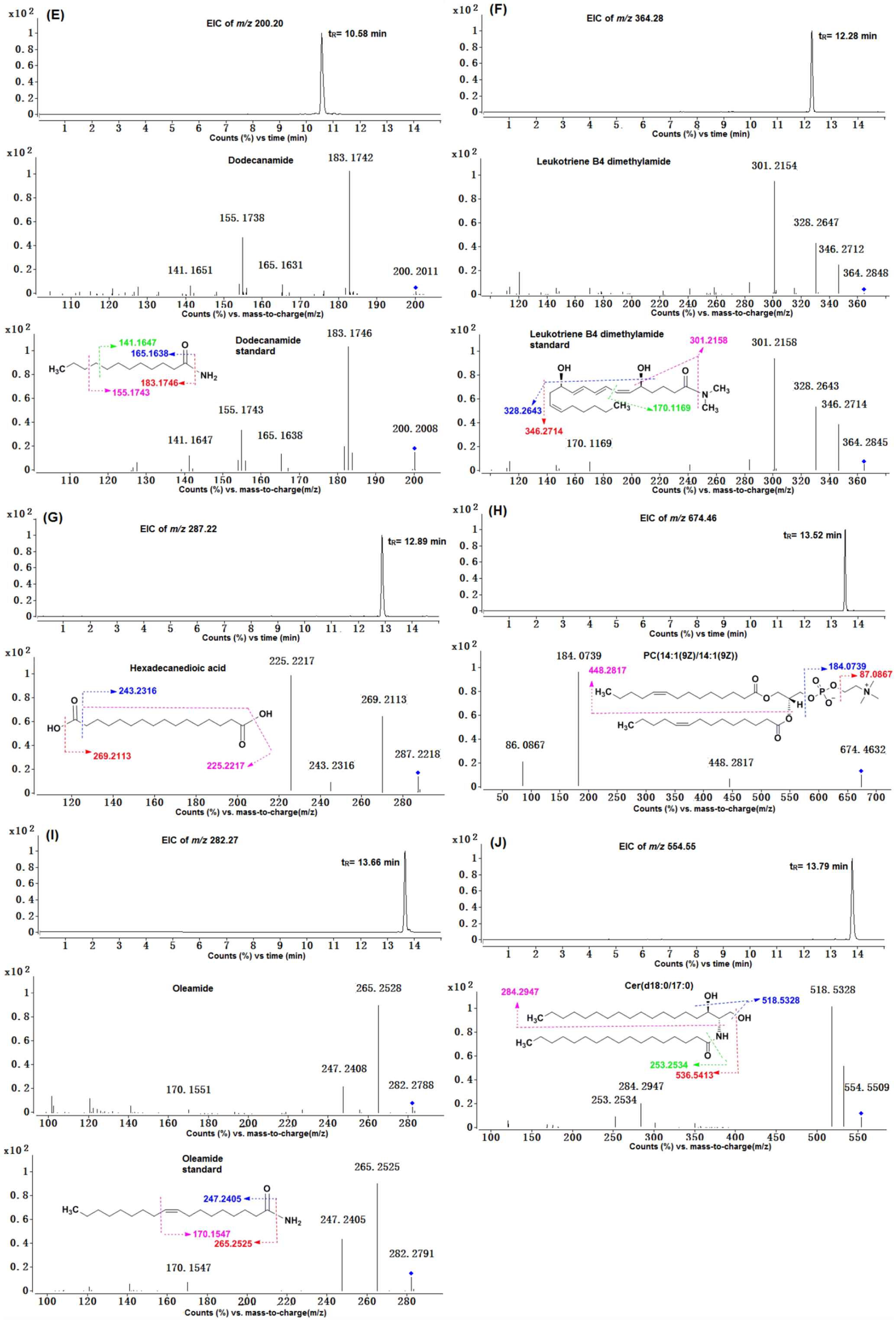

Supplementary Figure 2: Extracted ion chromatograms (EICs) and MS/MS mass spectra as well as predicted structures with expected fragmentation profiles of the ten metabolites in AML patient serum. **(A)** Hypoxanthine, **(B)** Phenylalanine, **(C)** Decanamide, **(D)** Sphingosine, **(E)** Dodecanamide, **(F)** Leukotriene B4 dimethylamide, **(G)** Hexadecanedioic acid, **(H)** PC(14:1(9Z)/14:1(9Z)), **(I)** Oleamide, and (G) Cer(d18:0/17:0) with or without comparison to commercially available standards.
